# Direct Degradation of Fresh and Dried Macroalgae by *Agarivorans albus* B2Z047

**DOI:** 10.3390/md22050203

**Published:** 2024-04-28

**Authors:** Ya Gong, Dan-Dan Shang, Cheng-Lin Sun, Zong-Jun Du, Guan-Jun Chen

**Affiliations:** 1 Marine College, Shandong University, Weihai 264209, China; gongya@sdu.edu.cn (Y.G.); duzongjun@sdu.edu.cn (Z.-J.D.); 2 State Key Laboratory of Microbial Technology, Institute of Microbial Technology, Shandong University, Qingdao 266237, China; 3 Weihai Research Institute of Industrial Technology, Shandong University, Weihai 264209, China

**Keywords:** *Agarivorans albus* B2Z047, macroalgae, *Saccharina japonica*, bioconversion, alginate lyase

## Abstract

Marine macroalgae are increasingly recognized for their significant biological and economic potential. The key to unlocking this potential lies in the efficient degradation of all carbohydrates from the macroalgae biomass. However, a variety of polysaccharides (alginate, cellulose, fucoidan, and laminarin), are difficult to degrade simultaneously in a short time. In this study, the brown alga *Saccharina japonica* was found to be rapidly and thoroughly degraded by the marine bacterium *Agarivorans albus* B2Z047. This strain harbors a broad spectrum of carbohydrate-active enzymes capable of degrading various polysaccharides, making it uniquely equipped to efficiently break down both fresh and dried kelp, achieving a hydrolysis rate of up to 52%. A transcriptomic analysis elucidated the presence of pivotal enzyme genes implicated in the degradation pathways of alginate, cellulose, fucoidan, and laminarin. This discovery highlights the bacterium’s capability for the efficient and comprehensive conversion of kelp biomass, indicating its significant potential in biotechnological applications for macroalgae resource utilization.

## 1. Introduction

Marine macroalgae, functioning as significant primary producers within coastal ecosystems, play a crucial role in sequestering carbon on a global scale, thereby acting as a vital component of the Earth’s carbon sink mechanisms [1]. Brown algae, including alginate-bearing *Saccharina japonica* and agar-bearing *Gracilaria*, are widely distributed around the world and sustain aquaculture extensively [2]. Asian countries, particularly China and Indonesia, with respective production outputs of 17.5 million tons and 9.7 million tons, have emerged as the predominant producers of marine macroalgae on a global scale [3]. Most marine macroalgae (70%) are utilized as a food source, while the remaining 30% find applications in various sectors, such as fertilizers, industrial products, medical products, cosmetics, and for producing biofuel [4,5]. Brown algae are enriched with carbohydrates, constituting approximately 35–60% of their dry weight, and their growth primarily requires sunlight and seawater [6,7,8]. Consequently, brown algae have attracted considerable interest as a viable renewable biomass resource for the generation of biofuels and chemicals.

*S. japonica* is one of the most important commercially farmed marine macroalgae globally [2]. It harbors several carbohydrates, including mannitol (the primary product of photosynthesis accumulation), laminarin (a storage polysaccharide), cellulose (a rigid fibrillar layer within the cell wall), alginate, and fucoidan (both extracellular polysaccharides and integral components of the cell wall matrix) [9]. Laminarin, a storage β-glucan of low molecular weight, predominantly comprises (1,3)-β-d-glucan [10]. Cellulose, a fibrous and water-insoluble polysaccharide, consists of d-glucose units joined through β-1,4-glycosidic bonds. Its content in *S. japonica* has been reported to be relatively low [11]. Alginate, the predominant polysaccharide, displays variations in content across different seasons, species, and subsections [12]. The content of alginate can escalate to constitute as much as 50% of the dry weight. Alginate is an acidic, polysaccharide formed through 1,4-linked glycosidic bonds that connect β-d-mannuronate and α-l-guluronate. Fucoidan, a kind of fucose-containing sulfated polysaccharide, exhibits a complex chemical composition with numerous structural variations observed both between species and during seasonal variations [13]. These different carbohydrates are primarily bound within polymers, rendering them less accessible to most microbes. However, notable advancements in the field have showcased the potential of directly fermenting marine macroalgae polymers to produce citramalate and fuels [14,15,16]. To enhance the comprehensive utilization of *S. japonica* and achieve increased value through cascading processes, it is crucial to overcome the recalcitrance of various macroalgal polysaccharides.

Most studies are primarily focused on the degradation of individual polysaccharide components following extraction, with limited attention given to the direct degradation of whole biomass, including both fresh and dried brown algae. Previous research has demonstrated that *Bacillus weihaiensis* Alg07 can grow by using kelp powder in the modified Marine Broth 2216 medium [17]. Additionally, *Tamlana* sp. S12 and *Pseudoalteromonas agarivorans* A3 have been found to decompose kelp pieces [18,19], while *Defluviitalea phaphyphila* Alg1 and *Vibrio* sp. Dhg can each be cultured anaerobically in the bioreactor and produce ethanol from the powder of dried kelp [5,15]. In addition, *Zobellia galactanivorans* Dsij has been observed to exhibit obvious growth, resulting in visible damage to fresh brown algae *Laminaria digitata* pieces [20]. These studies suggested the sequential utilization of various macroalgal polysaccharides present in the whole kelp. In this study, we employed *Agarivorans* sp. B2Z047, a highly efficient alginate-degrading bacterium [21], to degrade both fresh and dried kelp pieces, as well as the kelp powder. We measured the growth of strain B2Z047 and the hydrolysis rate of the kelp. Additionally, we investigated the optimal hydrolysis conditions. During the process of kelp decomposition, extracellular polysaccharidases, degradation products, and transcriptomic changes were analyzed to identify key genes specifically induced by the presence of the whole kelp biomass and to elucidate the potential mechanism.

## 2. Results and Discussion

### 2.1. Agarivorans albus B2Z047 Can Efficiently and Rapidly Degrade Kelp Pieces into Sludge

Strain B2Z047, isolated from the gut of adult abalone, exhibits efficient alginate degradation [21]. To establish its taxonomic classification, we conducted phylogenetic analyses using concatenated sequences of 120 ubiquitous single-copy proteins through GTDB pipelines (Figure S1). The genome tree revealed that the strains B2Z047 and *Agarivorans albus* JCM 21469^T^ clustered within the same clade, consistent with the ANI and dDDH values (Table S1). As a result, the strain B2Z047 was identified and classified as a new member within the *Agarivorans albus* species, hereby designated as *Agarivorans albus* B2Z047. The general features of strain B2Z047 are summarized in Table S2.

**Figure 1:**
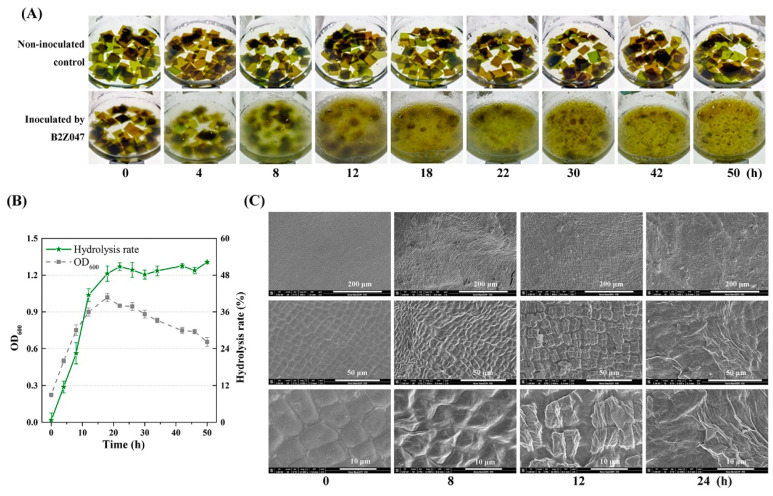
*A. albus* B2Z047 degrades brown algae *S. japonica* pieces efficiently. Dried kelp pieces (0.5 g) were incubated in BM medium with or without strain B2Z047. Following incubation at 25 °C, the morphology of *S. japonica* pieces (**A**), the growth of *A. albus* B2Z047 and the hydrolysis rate of *S. japonica* (**B**), and the SEM micromorphology of *S. japonica* pieces (**C**). Scale bars are 10, 50, and 200 μm.

Given its high-efficiency alginate degradation, *A. albus* B2Z047 was chosen for the assessment of *S. japonica* degradation and cultured in the kelp medium with a sole carbon source of *S. japonica*. The results demonstrated the efficient degradation of the *S. japonica* pieces by strain *A. albus* B2Z047 (Figure 1A). In the absence of *A. albus* B2Z047, the *S. japonica* pieces retained their complete 1.0 cm × 1.0 cm sheet structure at 50 h. Conversely, when *A. albus* B2Z047 was added, its observable growth occurred at 4 h, reaching a maximal cell concentration of 1.02 (OD_600_) at 18 h in the kelp medium (Figure 1B). The degradation of the lamellar structure of *S. japonica* was observed at 8 h, displaying irregular degradation and rupture. The majority of the lamellar *S. japonica* pieces were reduced to small particles at 18 h. As the time progressed, the cell density of strain B2Z047 increased, accompanied by the collapse of the lamellar *S. japonica* pieces. The hydrolysis rate reached approximately 50.8% at 22 h (Figure 1B). Importantly, it should be noted that the continuous extension of the culture time did not lead to a further improvement in the degradation rate, though the hydrolysis rate at 50 h was 52.3%. These results showed that strain *A albus* B2Z047 possessed an exceptional capability in efficiently and rapidly degrading the kelp fragments. In recent studies, it has been observed that *P. agarivorans* A3 has the ability to degrade pieces and the powder of dried kelp [19]. Additionally, certain strains from *Tamlana*, *Algibacter*, *Maribacter*, and *Zobellia* have been found to utilize dried kelp pieces for their growth [18]. Notably, *Tamlana* sp. S12 exhibited the highest cell density (OD_600_ ≈ 0.7) at 24 h, and the kelp pieces were visibly damaged at 48 h. Our findings highlight the unique and remarkable ability of this single strain in kelp degradation.

To elucidate the degradation characteristics, SEM was used to analyze the morphological alterations occurring in *S. japonica* pieces, subsequent to the incubation with *A. albus* B2Z047. Before fermentation, the SEM photographs revealed an intact and smooth cell wall structure of the *S. japonica* pieces (Figure 1C). When cultured with the strain B2Z047, a dramatic transformation occurred, disrupting the initial ordered and uniform cell structures, resulting in a loose and rugged appearance. Prolonged hydrolysis exposed more pores, culminating in collapsed and incomplete cell structures. Evidently, strain B2Z047 plays a pivotal role in degrading the cell wall components, mainly composed of cellulose, alginate, and fucoidan, thereby altering the cell morphology of the brown algae, *S. japonica*.

### 2.2. Optimization of Hydrolysis Conditions

**Figure 2:**
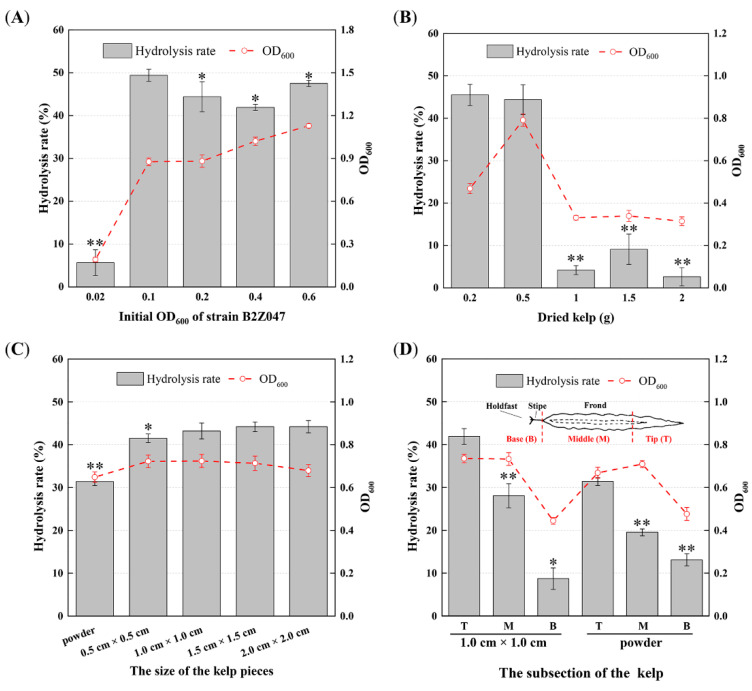
Optimization of degrading parameters of *S. japonica* by *A. albus* B2Z047; 1% dried *S. japonica* was added into artificial seawater to optimize seed liquid inoculation (**A**) and the weight (**B**), size (**C**), and subsection (**D**) of *S. japonica* (* *p* < 0.05, ** *p* < 0.01).

The initial OD_600_ of strain B2Z047 as well as the weight, size, and subsection of the kelp were optimized to enhance the hydrolysis rate of the *S. japonica* tissues. Adjusting the initial OD_600_ of strain B2Z047 from 0.02 to 0.1 in the kelp medium led to a significant increase in the hydrolysis rate, elevating it from 5.67% to 49.40% (Figure 2A). When the weight of *S. japonica* changed from 0.2 g and 0.5 g to 1.0 g, the hydrolysis rate exhibited a significant decline from 45.5% to 4.2% (Figure 2B). It is conceivable that the dried *S. japonica* pieces absorbed excessive water in the medium, leading to heightened viscosity, which could potentially inhibit bacterial growth. This hypothesis was supported by the experimental results, as the cell concentrations of strain B2Z047 were below 0.4 OD_600_ in the shaken flask cultures containing 1.0 g, 1.5 g, and 2.0 g of the dried kelp pieces. Concerning the optimization of the kelp piece size, no significant difference was observed in the hydrolysis rate when the diameter of the pieces was varied (Figure 2C). However, the hydrolysis rate in the kelp powder was approximately 31.4%, which was significantly lower than that observed in the kelp pieces. This result might be attributed to the increased viscosity in the kelp powder medium.

To compare the hydrolysis variance among distinct structural components of *S. japonica*, the tip frond, the middle frond, and the base (holdfast and stipe) were individually subjected to 24 h of hydrolysis at 25 °C. The hydrolysis rates were 41.9%, 28.07%, and 8.73%, respectively (Figure 2D). This difference may be attributed to variations in the component content within the different segments of *S. japonica*. Specifically, the composition of the base was high in cellulose and alginate, which are not easily degradable, whereas the tip frond harbored more mannitol and laminarin [12]. Additionally, different segments of fresh kelp, without any processing, were hydrolyzed by strain B2Z047, yielding similar results (Figure S2), and was faster than that of *Z. galactanivorans* Dsij [20]. We also observed that the hydrolysis rate of the powdered *S. japonica* was generally lower than that of its flaky structure (Figure 2D). Consequently, in the follow-up experiments, the conditions of an initial OD_600_ of 0.1, a dry weight of 0.5 g, and kelp pieces of 1.0 cm × 1.0 cm were determined as the most suitable for strain B2Z047 to effectively hydrolyze the brown alga *S. japonica* in 50 mL of artificial seawater.

### 2.3. The Extracellular Polysaccharidases of Strain B2Z047 during Kelp Decomposition

**Figure 3:**
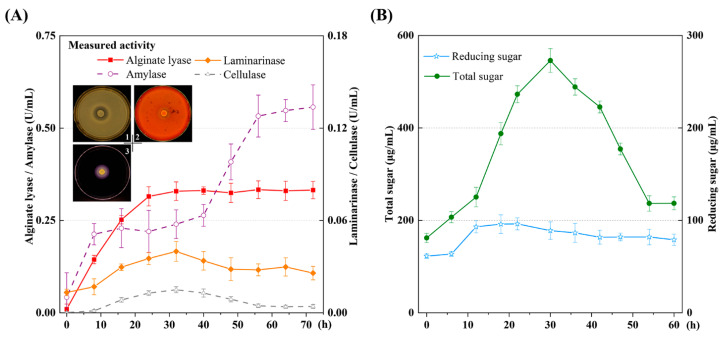
Extracellular polysaccharidases and products during the degradation of *S. japonica*. (**A**) The enzyme activity levels were detected every 8 h. (**B**) The reducing sugar and total sugar curves in the kelp medium were analyzed to assess the overall polysaccharide degradation.

Due to the diverse polysaccharide components present in the brown alga *S. japonica*, a comprehensive analysis of the extracellular polysaccharidases was conducted to identify the potential key enzymes involved in kelp degradation. Firstly, the polysaccharidases were observed on plates containing sodium alginate, starch, or carboxymethyl cellulose (Figure 3A). Subsequently, the enzyme activities were measured during the degradation of the brown alga *S. japonica*. Notably, the alginate lyases and amylases displayed a swift upsurge within the initial 8 h, whereas the laminarinases and cellulases demonstrated an increase after this period. The activity of the alginate lyases exhibited a sustained rise, reaching its peak of approximately 0.33 U/mL at 32 h. The cellulases and laminarinases demonstrated a moderate culmination, reaching peaks of approximately 0.02 and 0.04 U/mL, respectively, at 32 h. However, the activity of the amylases experienced a secondary rapid increase between 40 and 56 h, attaining its maximum value of 0.56 U/mL at 72 h. This result might be attributed to the functionality of cellulases, resulting in the release of laminarins and fucoidans into the medium. Hence, *A. albus* B2Z047 exhibited the capability to produce a range of polysaccharidases, with these enzymes synergistically cooperating to facilitate the rapid and efficient hydrolysis of the brown alga *S. japonica*.

To further confirm the degradation of the kelp carbohydrates, the contents of total sugar and reducing sugar were measured in the fermentation broth of strain B2Z047 cultured with *S. japonica*. The total sugar content was determined by using the phenol–sulfuric acid method [22]. The total sugar increased slowly within the initial 12 h, followed by a rapid surge between 12 and 30 h, reaching a peak of 545.79 μg/mL at the 30 h mark (Figure 3B). This value aligned with the overall polysaccharide content of the brown alga *S. japonica*, constituting approximately 40.8–67% (*w/w*) of its dry weight [23]. In this study, 0.5 g of the dried kelp pieces was added to 50 mL of artificial seawater, yielding a total carbohydrate concentration ranging from 408 to 670 μg/mL. Subsequently, the total sugar gradually decreased, with the final residues measuring approximately 236.61 μg/mL. The content of reducing sugar consistently remained at a low level throughout the entire process of *S. japonica* degradation. However, an exception occurred with an increase observed between 6 and 12 h, reaching a peak at 96.21 μg/mL at 22 h. Mannitol was rapidly exhausted in less than 8 h (Figure S3). Combined with the SEM findings during *S. japonica* degradation, the activities of extracellular polysaccharidases, along with the total sugar and reducing sugar contents, indicated that the polysaccharides were likely released through the lysis of kelp cells. Consequently, the total sugar in the fermentation broth increased, undergoing subsequent degradation by alginate lyases, amylases, laminarinases, and cellulases. This process coincided with the growth of strain B2Z047, which probably contributed to the degradation and conversion of these carbohydrate products. As a result, both the total sugar and reducing sugar contents ultimately decreased.

### 2.4. The Transcriptomic Profile of Diverse Carbohydrate-Degrading Enzymes

The CAZys presented in strain B2Z047 were identified by utilizing the dbCAN3 server, employing more than two tools [24]. The comprehensive analysis revealed a total of 12 polysaccharide lyases, 113 glycoside hydrolases, 47 carbohydrate-binding modules, and 44 glycosyl transferases (Table S3). Among these, the alginate lyase sequences were classified into four polysaccharide lyase (PL) families, namely, PL6 (1), PL7 (8), PL17 (2), and PL38 (1). Previous studies have documented the function of these genes, and the degradation products of alginate have been successfully detected [21]. The sequence analyses further revealed that 10 alginate lyase genes were shared within the *Agarivorans* genus, while the PL7 alginate lyase-encoding genes *aly7A1* (*LQZ07_RS04225*) and *aly7A4* (*LQZ07_RS14510*) were potentially gained from *Vibrio* via horizontal gene transfer. Moreover, the genome of strain B2Z047 was found to contain 41 glycoside hydrolase (GH) families, with the main members belonging to families GH13 (22), GH16 (10), and GH5 (9). These glycoside hydrolases exhibit activity towards glycosidic bonds in various complex polysaccharides (http://www.cazy.org/, [25]). As a result, strain B2Z047 exhibits the potential to degrade various polysaccharides, such as cellulose (GH5, GH8, and GH9), fucoidan (GH1, GH3, and GH141), laminarin (GH13, GH16, and GH17), and agarose (GH50, GH86, and GH118). These findings are corroborated by the observed polysaccharide degradation on the plates and the corresponding enzyme activities during kelp decomposition (Figure 3A).

**Figure 4:**
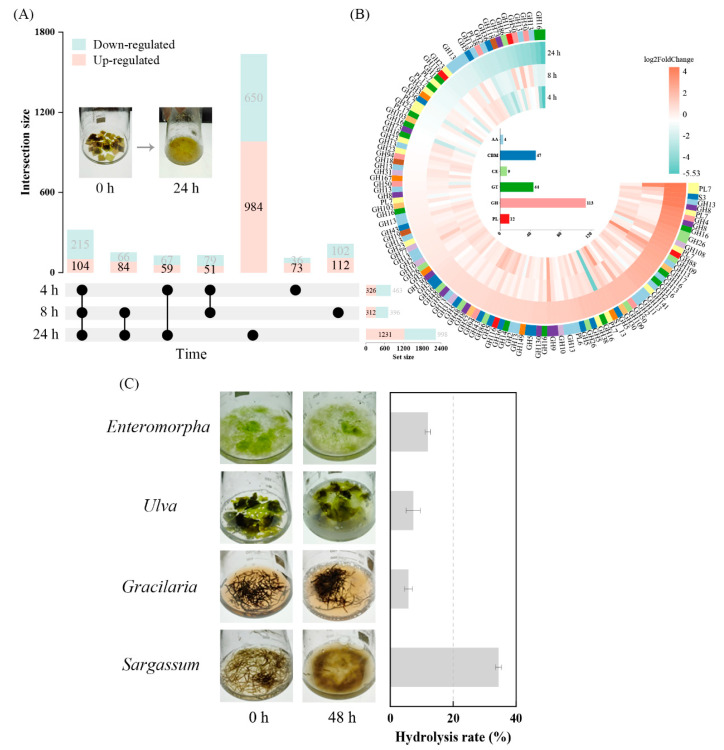
Transcriptomic responses of *A. albus* B2Z047 during the process of kelp decomposition. (**A**) Differentially expressed genes were illustrated in the upset plot, with 0 h sample as the control. The total amount of genes regulated at each time point is shown in the set size. (**B**) Heatmap of all 113 glycoside hydrolases, 12 polysaccharide lyases, and 2 sulfatases identified in the genome of *A. albus* B2Z047. The log_2_FoldChange for each gene is indicated, taking 0 h sample as the control. (**C**) Degradation capabilities of *A. albus* B2Z047 towards various macroalgae species. Dried brown (*Sargassum*), green (*Enteromorpha* and *Ulva*), and red (*Gracilaria*) macroalgae were incubated with and without B2Z047 in artificial seawater. After 48 h incubation, the hydrolysis rate was measured.

To systematically identify the CAZy genes involved in the kelp degradation process, a transcriptome analysis of *A. albus* B2Z047 was conducted after 0 h, 4 h, 8 h, and 24 h of incubation. The mapping of the sequenced reads to the genome of *A. albus* B2Z047 yielded successful mapping rates ranging from 92.07% to 98.54%. The differential abundance analysis identified a total of 1467 upregulated and 1215 downregulated genes, using 0 h as a control (Data S1). Notably, a significant proportion of these genes, 67% (984 upregulated genes) and 53% (650 downregulated genes), exhibited specific regulations at the 24 h time point (Figure 4A). Moreover, we identified a core set of genes that displayed consistent expression patterns across all three degradation time points, consisting of 104 upregulated genes and 215 downregulated genes. The hierarchical clustering of the gene expressions of all 113 glycoside hydrolases, 12 polysaccharide lyases, and 2 sulfatases in the *A. albus* B2Z047 genome revealed that most of the genes were significantly induced with the kelp at different time points (Figure 4B). Among them, 23 genes were induced at least 4-fold at 24 h. Four genes belonging to the PL7 alginate lyase family exhibited a significant induction of at least 7-fold, with notable upregulation observed of *aly7A1* (*LQZ07_RS04225*, 21.7-fold) and *aly7A3* (*LQZ07_RS11580*, 20.8-fold). In addition, the induced genes *LQZ07_RS06905* (18.1-fold, GH13), *LQZ07_RS07330* (18-fold, GH8), and *LQZ07_RS24045* (4.7-fold, GH141) had the potential to encode enzymes capable of degrading laminarin, cellulose, and fucoidan, respectively. Two genes encoding sulfatases, *LQZ07_RS17175* (19.3-fold, S3) and *LQZ07_RS06285* (3.6-fold, arylsulfatase S1_13), were also upregulated, indicating their potential involvement in the degradation of sulfated polysaccharide substrates. Furthermore, *A. albus* B2Z047 was employed for the degradation of various types of macroalgae, including brown (*Sargassum*), green (*Enteromorpha*, *Ulva*), and red (*Gracilaria*) macroalgae (Figure 4C). Notably, the findings revealed that strain B2Z047 exhibited the capability to rapidly degrade brown algae.

## 3. Materials and Methods

### 3.1. Experimental Materials

The brown alga *S. japonica* was harvested in April 2021 from farms located in Weihai, China (37.26° N, 122.61° E). The harvested *S. japonica* was washed three times to eliminate any traces of dirt and salt, followed by sun drying. Subsequently, the dried *S. japonica* was either cut into pieces or milled into powder for further use. Sodium alginate was obtained from Bright moon seaweed group Co., Ltd., (Qingdao, China). Standard sodium carboxymethyl cellulose, laminarin, glucose, and mannitol were obtained from Sigma (St. Louis, MO, USA).

### 3.2. S. japonica Degradation Assay with Strain B2Z047

Strain B2Z047 used in this study was isolated from the gut of adult abalone and was deposited in the culture collection in China (CGMCC No. 22885). The basal medium (BM) employed in the experiment consisted of 0.5% peptone, 0.2% sodium pyruvate, and 0.1% yeast extract, which were dissolved in artificial seawater containing 0.32% MgSO_4_, 0.22% MgCl_2_, 0.12% CaCl_2_, 0.07% KCl, and 0.02% NaHCO_3_ (*w/v*). The kelp medium was prepared as 1% kelp pieces or powder in artificial seawater.

Strain B2Z047 was initially inoculated into a 10 mL basal medium (BM) culture and maintained at 25 °C, 175 rpm. Subsequently, a 1% seed culture was transferred into a 50 mL BM medium during the exponential growth phase. In a separate 250 mL Erlenmeyer flask, 50 mL kelp medium was prepared and autoclaved. The seed solution of B2Z047 was centrifuged (5 min at 8000 rpm) to remove the supernatant, after which the bacteria were resuspended in the kelp medium. Regular intervals were observed, and images were captured to monitor the degradation of *S. japonica*. Additionally, the hydrolysis rate was calculated, and changes in B2Z047 biomass were detected.

To collect *S. japonica* pieces, the culture in the flask was passed through a 200-mesh sieve. The collected pieces were dried at 72 °C overnight. The hydrolysis rate of *S. japonica* was quantified utilizing the following equation [26]:

$$Hydrolysis\;rate\left(\%\right)=\left(1-\frac{W_{R}}{W_{S}}\right)\times 100$$

Here, *W_S_* represents the weight of the *S. japonica* sample on a dry basis, while *W_R_* corresponds to the dry weight of the recovered *S. japonica* sample after inoculation with strain B2Z047.

Furthermore, 2 mL of the filtrate was subjected to a low-speed brief centrifugation (30 s at 3000 rpm) to remove any remaining kelp pieces. Following this step, 200 μL of the upper layer containing bacteria was slowly absorbed into a 96-well plate for measuring cell concentration (OD_600_). All experiments were performed in triplicate.

### 3.3. Microscopic Observation of Kelp Pieces

The morphology of *S. japonica* samples that were both hydrolyzed and non-hydrolyzed for varying periods of time was analyzed using a scanning electron microscope (SEM; Nova NanoSEM 450, FEI, Hillsboro, OR, USA). The *S. japonica* samples were subjected to three washes with phosphate buffer. Subsequently, they were immersed in a 2.5% glutaraldehyde solution for more than 4 h. Following fixation, the samples underwent a dehydration process utilizing a graded series of ethanol concentrations. To facilitate complete removal of water, a critical-point dryer was employed. The samples were then coated with gold prior to observation.

### 3.4. Analytical Methods for Products and Enzyme Activities

The quantification of total sugar content was carried out employing the phenol–sulfuric acid assay, with absorbance measurements taken at a wavelength of 490 nm. Standard curves were generated using a series of alginate solutions. The change in reducing sugar within the kelp fermentation broth was determined employing the 3,5-dinitrosalicylic acid assay, utilizing glucose as the calibration standard. To determine the mannitol content, a colorimetric method was employed using mannitol as the standard [27]. Briefly, the sample was mixed with sodium periodate (5 mM) and formate (0.5 M, pH 3.0) in a 1:3:5 ratio, and then we added a third volume of a reaction buffer containing thiosulfate (20 mM), acetylacetone (100 mM), and ammonium acetate (2 M). The tube was then closed and subjected to heating within a boiling water bath for 2 min. Upon cooling, the absorbance was detected at 412 nm.

The marine agar 2216 media were prepared, containing sodium alginate, starch, and sodium carboxymethyl cellulose (0.5%, *w/v*), respectively. Subsequently, a 2 μL suspension of *A. albus* B2Z047 in the exponential growth phase was added dropwise to the plate. After 3 days of cultivation at 25 °C, the activities of alginate lyase, amylase, and cellulase were detected using 1% CaCl_2_ solution [28], Lu’s iodine solution, and Congo red solution [29], respectively.

To evaluate the activities of alginate lyase and amylase, the supernatant obtained from the degradation process of *S. japonica* was combined with soluble substrates (0.5% alginate/starch) in a 1:9 ratio [30,31]. This mixture was then incubated at 40 °C for 15 min. Similarly, for cellulase and laminarinase activities, the fermentation supernatant and substrates were mixed in a 1:1 ratio. The absorbance of the reaction mixture was determined at 540 nm. The kelp medium without B2Z047 was used as a blank control in the assay. One unit of enzyme activity was defined as the amount of enzyme that can produce 1 mg of reducing sugar equivalent to 1 mg of glucose per minute.

### 3.5. Genome Analysis and Annotation

The annotation of carbohydrate-active enzymes (CAZys) was conducted through the application of the dbCAN3 server with default settings [24]. For the assignment of enzymes to specific CAZy families, predictions made by a minimum of two out of the three algorithms (dbCAN_sub, HMMER, and DIAMOND) were deemed adequate. Sulfatases were identified using the version 2.3.1 database [32,33]. The phylogenetic relationships among the species were investigated by analyzing amino acid sequences using GTDB-Tk. Phylogenetic trees were constructed using FastTree and IQTree with 1000 bootstrap replicates. The EzGenome service and the Genome-to-Genome Distance Calculator were employed for calculation of the average nucleotide identity (ANI) and the digital DNA–DNA hybridization (dDDH) [34].

### 3.6. Transcriptomic Analysis

Using a conventional extraction protocol, total RNA was isolated from *A. albus* B2Z047. The isolated RNA was evaluated through 1% agarose gel electrophoresis. Additionally, an Agilent 2100 system was utilized to evaluate the integrity and total amount of RNA. The sequencing library was generated according to the strand-specific library construction method [35]. Subsequently, Illumina sequencing was executed at the Novogene Bioinformatics Technology Co., Ltd., with sample pooling guided by specific requirements for effective concentration and targeted data volume. The raw data underwent filtration to exclude reads of low quality, poly-N sequences, and those containing an adapter. Gene transcription levels were quantified employing the RPKM algorithm. Differential expression analysis was performed using DESeq, identifying genes as differentially expressed (adjusted *p*-value < 0.05). The upset plot and heatmap were generated using ChiPlot (https://www.chiplot.online/ accessed on 20 December 2023).

## 4. Conclusions

This study revealed that *S. japonica* can be efficiently degraded by strain B2Z047, with a 52% hydrolysis efficiency, offering an eco-friendly alternative to traditional hydrolysis methods. The transcriptomic analysis revealed an upregulation of the genes for key enzymes, including alginate lyase, laminarinase, cellulase, and amylase, enabling the complete breakdown of the kelp carbohydrates. Additionally, *A. albus* B2Z047’s complex regulatory system for polysaccharide utilization suggests a promising strategy for transforming macroalgal biomass into valuable resources, enhancing the sustainable exploitation of marine biological materials.

## Data Availability

The complete genome of strain B2Z047 have been deposited at DDBJ/ENA/GenBank under the accession number CP088080.

## Supplementary Materials

The following supporting information can be downloaded at: https://www.mdpi.com/article/10.3390/md22050203/s1, Figure S1. Phylogenomic tree based on alignment of 120 conserved proteins showing the taxonomic position of strain B2Z047. Figure S2. *A. albus* B2Z047 degrades the fresh brown alga *S. japonica* efficiently. Figure S3. The mannitol curves during the degradation of *S. japonica*. Table S1. GGDC (%) values (dDDH) and ANI values among the genomes of strain B2Z047 and the related strains of the genus *Agarivorans*. Table S2. General features and genome sequencing information of *A. albus* B2Z047. Table S3. Distribution of CAZyme classes count in strain B2Z047. Data S1. Carbohydrate-active enzyme gene expression of strain B2Z047 at different time points: 0 h, 4 h, 8 h, and 24 h.
