# Supplementary material for: Direct Degradation of Fresh and Dried Macroalgae by Agarivorans albus B2Z047
Source: Mar Drugs. 2024 Apr 28;22(5):203. doi: 10.3390/md22050203 (PMC11122777; doi:10.3390/md22050203)
Supplement: Supplementary file 1 [file marinedrugs-22-00203-s001.zip › Supplementary materials.pdf]

Supplementary materials

Direct degradation of fresh and dried macroalgae by *Agarivorans albus* B2Z047

Ya Gong[1,2,3], Dan-Dan Shang[1], Cheng-Lin Sun [1], Zong-Jun Du[1,2,3] and Guan-Jun Chen[1,2,*]

[1]Marine College, Shandong University, Weihai 264209, China.
[2]State key Laboratory of Microbial Technology, Institute of Microbial Technology, Shandong University, Qingdao 266237, China.
[3]Weihai Research Institute of Industrial Technology of Shandong University, Weihai 264209, China

[*]Correspondence: guanjun@sdu.edu.cn

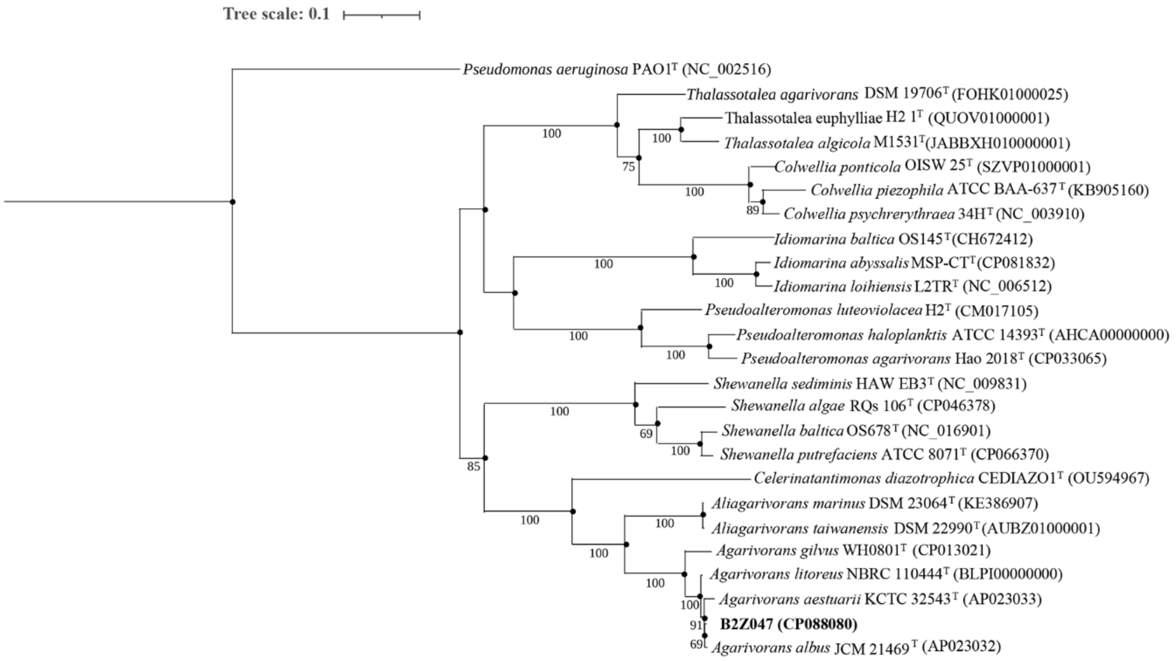

Figure S1. Phylogenomic tree based on alignment of 120 conserved proteins showing the taxonomic position of strain B2Z047. Numbers on nodes represent bootstrap values based on 1000 replications. Bootstrap values above 70% (based on 1000 replicates) are shown at branch nodes. Bar, 0.1 substitutions per nucleotide position.

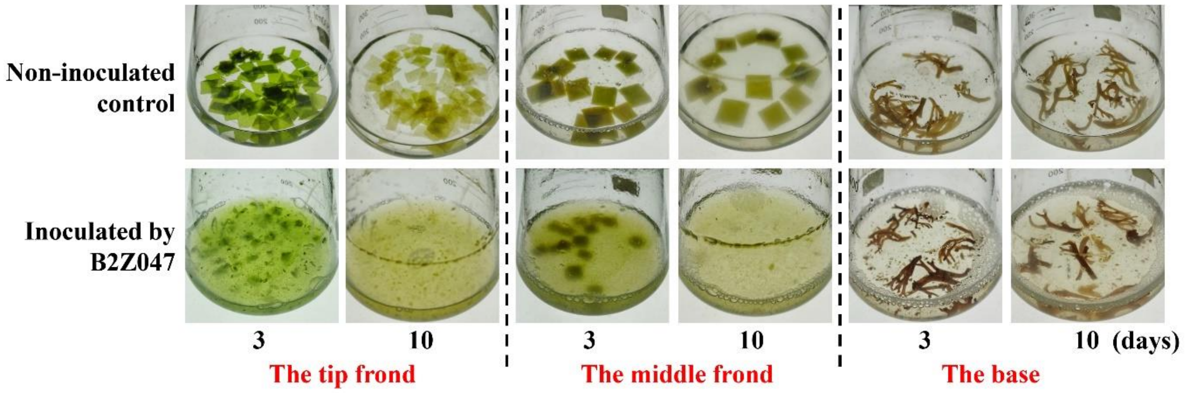

Figure S2. *A. albus* B2Z047 degrade fresh brown algae *S. japonica* efficiently.

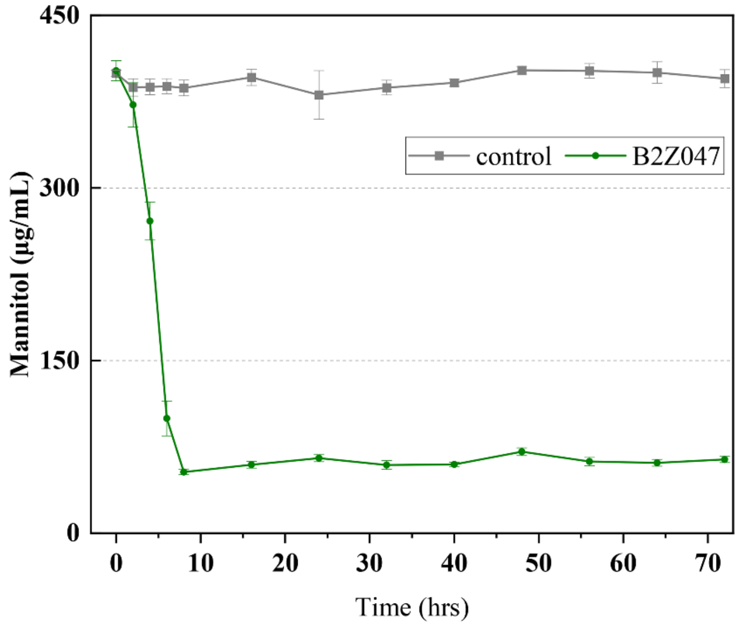

Figure S3. The mannitol curves during the degradation of *S. japonica*. The kelp medium without B2Z047 was used as the control.

Table S1. GGDC (%) values (dDDH) and ANI values among the genomes of strain B2Z047 and the related strains of the genus *Agarivorans*.

**ANI** / **dDDH**	**B2Z047**	***Agarivorans albus*** **JCM 21469^T**	***Agarivorans gilvus*** **WH0801^T**	***Agarivorans aestuarii*** **KCTC 32543^T**	***Agarivorans litoreus*** **NBRC 110444^T**
**B2Z047**	-	96.52	79.23	90.70	91.96
Agarivorans albus **JCM 21469^T**	75.80	-	79.29	91.38	92.61
Agarivorans gilvus **WH0801^T**	26.10	19.90	-	79.38	79.59
Agarivorans aestuarii **KCTC 32543^T**	48.00	44.40	20.10	-	89.29
Agarivorans litoreus **NBRC 110444^T**	52.60	49.50	20.40	38.20	-

Table S2. General features and genome sequencing information of *A. albus* B2Z047.

Property	Term
**General features**	
Classification	Domain *Bacteria*<br>Phylum *Proteobacteria*<br>Class *Gammaproteobacteria*<br>Order *Alteromonadales*<br>Family *Alteromonadaceae*<br>Genus *Agarivorans*<br>Species *Agarivorans albus*<br>Strain B2Z047
Gram stain	Negative
Cell shape	Short rod
Motility	Flagella motility
Temperature range; optimum	4-37 °C; 30 °C
pH range; optimum	6.0-10.0; 7.0-8.0
Salinity, optimum	1.0–8.0 % NaCl (w/v); 3.0 %
Oxygen requirement	Aerobic
**Sequencing**	
Sequencing platform	PacBio SMRT
Fold coverage	332 ×
Assembly method	HGAP4/Falcon
**Genome features**	
Genome size (bp)	5,298,843 bp
G+C content (%)	44.14 %
Protein-coding sequences (CDS)	4853
Gene total length of coding gene (bp)	4,648,614 bp
Average CDS length (bp)	957.88
rRNA	22
tRNA	93
Other ncRNA	46
GenBank serial number	CP088080
16S rRNA gene serial number	OM278383
NR annotation number (percent%)	4681 (96.46)
COG annotation number (percent%)	3986 (82.13)
GO annotation number (percent%)	2744 (56.54)
KEGG annotation number (percent%)	1518 (31.28)
CAZyme annotation number (percent%)	197 (4.06)

Table S3. Distribution of CAZyme classes count in strain B2Z047.

CAZy class	Count	CAZy class	Count	CAZy class	Count
AA2	1	GH1	1	GH94	2
AA3	1	GH2	5	GH103	2
AA6	1	GH3	2	GH108	1
AA10	1	GH4	1	GH109	2
CBM2	9	GH5	9	GH116	1
CBM3	2	GH8	4	GH117	1
CBM4	5	GH9	3	GH118	1
CBM6	6	GH10	2	GH119	1
CBM20	1	GH11	1	GH130	2
CBM21	1	GH13	22	GH141	1
CBM22	2	GH14	1	GH149	1
CBM25	1	GH16	10	GH167	1
CBM26	1	GH17	1	GT1	1
CBM27	2	GH18	1	GT2	15
CBM31	1	GH19	1	GT4	12
CBM32	2	GH23	6	GT5	1
CBM35	2	GH26	4	GT9	5
CBM41	2	GH30	1	GT19	1
CBM48	2	GH31	2	GT28	1
CBM50	4	GH36	4	GT30	1
CBM59	1	GH38	1	GT35	1
CBM69	1	GH43	2	GT51	3
CBM73	1	GH48	2	GT70	1
CBM91	1	GH50	4	GT81	1
CE1	2	GH52	1	GT83	1
CE4	4	GH73	2	PL6	1
CE7	1	GH77	1	PL7	8
CE9	1	GH86	2	PL17	2
CE11	1	GH88	1	PL38	1
